# Effect of valdecoxib pretreatment on pain and secondary hyperalgesia: a randomized controlled trial in healthy volunteers [ISRCTN05282752, NCT00260325]

**DOI:** 10.1186/1471-2253-6-3

**Published:** 2006-03-10

**Authors:** David Burns, Lindsay Hill, Michael Essandoh, Tomasz M Jarzembowski, H Gregg Schuler, Piotr K Janicki

**Affiliations:** 1 Department of Anesthesiology, Pennsylvania State College of Medicine and Milton S. Hershey Medical Center, Hershey, Pennsylvania, USA

## Abstract

**Background:**

Induction of the COX-2 isoenzyme appears to play a major role in the genesis of central sensitization after nociceptive stimulation. This study aimed to investigate the efficacy of a single, oral dose of the specific COX-2 inhibitor-valdecoxib in attenuating the central sensitization – induced secondary hyperalgesia in a heat/capsaicin pain model in healthy volunteers.

**Methods:**

The study was a randomized, double blind, placebo controlled, crossover, single dose efficacy trial using 20 healthy volunteers. Two hours following placebo or 40 mg, PO valdecoxib, participants underwent skin sensitization with heat/capsaicin, as well as supra-threshold pain and re-kindling measurements according to an established, validated pain model. Subjects rated pain intensity and unpleasantness on a visual analog scale and the area of secondary hyperalgesia was serially mapped.

**Results:**

The area of secondary hyperalgesia produced after 40 mg of valdecoxib was no different than that after placebo. Furthermore, there were no significantly relevant differences when volunteers were treated with valdecoxib or placebo in relation to either cold- or hot pain threshold or the intensity of pain after supra-threshold, thermal pain stimulation.

**Conclusion:**

We demonstrated that a single, oral dose of valdecoxib when does not attenuate secondary hyperalgesia induced by heat/capsaicin in a cutaneous sensitization pain model in healthy volunteers.

## Background

Induction of the COX-2 isoenzyme appears to play a major role in the genesis of central sensitization after nociceptive stimulation, with combined central and peripheral COX-2 inhibition acting synergistically to reduce the excitatory consequences of nociceptive input [1,2]. The selective COX-2 inhibitors may offer significant advantages over existing non-selective COX inhibitors by enabling inhibition of the central sensitization component of secondary hyperalgesia, in addition to their peripheral analgesic activity [3-8]. Several COX-2 inhibitors including valdecoxib reach the central nervous system in humans, with rapid penetration, and in concentrations apparently sufficient to inhibit COX-2 activity [9].

To study the efficacy of valdecoxib in attenuating secondary hyperalgesia, we used the cutaneous heat-capsaicin sensitization model. This model is well validated and accepted for assessing secondary hyperalgesia and the efficacy of analgesic medications and has been extensively described [10-14]. The synergistic combination of non-invasive physical (heat) and chemical (capsaicin) mechanisms of nociceptor stimulation produces a relatively stable and long-lasting hyperalgesia with a low potential for skin injury [15]. Both the heat and capsaicin act by activating nociceptors in the skin [15]. Rekindling is the key step in the model that ensures continuous secondary hyperalgesia by providing a constant afferent signal from the site of primary injury [10].

The specific aim of this study was to investigate the effect of pretreatment with the COX-2 specific inhibitor valdecoxib on the development of the secondary hyperalgesia in humans. The hypothesis to be tested is that selective COX-2 inhibitors significantly attenuate the central component of sensitization, thereby decreasing the area of secondary hyperalgesia induced by our heat/capsaicin pain model.

## Methods

This prospective, randomized, double-blinded study was performed on 20 healthy adult volunteers (10 men and 10 women) using a crossover design (a single, oral dose of placebo vs. valdecoxib 40 mg) separated by a minimum one-week washout period. Subject treatment was determined according to a randomization scheme restricted by gender and prepared by our department of Health Evaluation Sciences and conveyed to our Investigational Drug Service without communication to any of the investigators. On the day of testing either placebo or active drug was dispensed by the pharmacist in envelopes marked with the subject name and treatment number (1 or 2) to one of the investigators. No treatment assignments were reveled to the investigators until after the last subject completed the protocol and all data had been collected. To further insure blinding subjects and investigators were asked to guess which treatment they received on a particular day. Analysis of these guesses was not statistically highly correlated with the actual treatment assignments. All clinical components of the study were performed in the clinical suites of the General Clinical Research Center (GCRC) at the Penn State Milton S. Hershey Medical Center. The Institutional Review Board approved the experimental protocol and Informed Consent form prior to initiation of the study.

All experimental sessions began at 7:00 am, following verification of Informed Consent, a negative pregnancy test, and baseline vital signs. Hot and cold pain thresholds were measured as well as the response to supra-threshold pain stimuli were obtained at baseline. After baseline measurements were obtained the subjects were given either active drug or placebo orally. Measurements of hot/cold pain thresholds were obtained 110 minutes after study substance or placebo administration, followed by initial heat-sensitization and application of capsaicin. The area of secondary hyperalgesia (HA), as well as the hot and cold pain threshold was measured at 160, 200, 240, 300, 340 and 400 minutes after placebo or study drug administration. The response to the supra-threshold pain stimulation was measured at 110 and 400 minutes after placebo or study drug administration. To evaluate patient blinding to study treatment, subjects were asked after each experimental session; to indicate which treatment they believed they had received (valdecoxib or placebo).

### Hot/cold pain threshold measurements

The pain threshold induced by hot and cold stimuli was determined by increasing the stimulus level until the sensation was perceived as painful, at which time the subject stopped the stimulus. Thermal stimulation was conducted using a 9 cm^2 ^thermode on the subject's non-dominant forearm. The thermode is controlled by a computerized regulator that starts at 32°C then warms or cools the surface of the thermode at a linear rate of 1°C/sec with a safety cutoff of 50°C or 0°C, for heat and cold, respectively (TSAII NeuroSensory Analyzer from Medoc Advanced Medical Systems, U.S., Minneapolis, MN). The subjects were instructed to terminate the heating or cooling at the initial perception of pain at which point the thermode temperature would return to baseline. Two series of cold and then hot thresholds were measured with four stimuli in each series and the average of the eight trials recorded.

### Supra-threshold pain measurements

Supra-threshold pain measurements were also performed on the medial aspect of the non-dominant forearm. Supra-threshold pain measurements were initiated at 32°C and progressed linearly at 1°C /sec. to a maximum of 48°C and then held there for 5 seconds. This session could as well be terminated at any time if the subject perceived the stimulus unacceptably painful or unpleasant. Study subjects were asked to report the level of perceived pain intensity (algosity) and unpleasantness by means of a 100 mm visual analog scale (VAS) for each pain aspect immediately after each stimulus. Before starting all measurements, an explanation was provided regarding the differences between the two aspects of pain: namely algosity (the sensory dimension of pain intensity) and unpleasantness (the emotional dimension of pain). To illustrate the differences between the domains, a loud noise analogy was used: the volume of the music represents the painfulness, and the obtrusiveness of the music represents the unpleasantness. The VAS ranged from "no pain" at one end to "the worst pain imaginable" at the other end. For measurement of unpleasantness, the wording was changed in accordance with "no unpleasantness" on one end and "the most unpleasant feeling" on the other end. The results were the average of two stimulation sessions.

### Cutaneous sensitization and rekindling

Cutaneous sensitization was performed by heating the skin on the volar aspect of the dominant forearm to 45°C for 5 minutes. Immediately thereafter, 0.5 g of capsaicin cream 0.075% (Capsagesic-HP) was applied to the heated area of skin and covered with a transparent dressing. After 30 minutes the dressing and capsaicin was removed. To ensure continuous secondary hyperalgesia, skin sensitization was rekindled (RK) 70 minutes after the initial application of the thermode, and at 40-minute intervals thereafter. Rekindling was achieved by reheating the previously sensitized skin to 40°C for 5 minutes.

### Secondary hyperalgesia (HA) measurements

Assessment of hyperalgesia was performed on the volar aspect of the dominant forearm after initial thermode/capsaicin skin sensitization as well as after subsequent rekindling. After heat stimulation the area of secondary hyperalgesia was quantified with a foam brush (allodynia) and a 26 g von Frey hair (mechanical hyperalgesia) by stimulating the skin distant from the treated area and slowly moving inward until the subject indicated the stimulus had become noxious or until the subject reported a definite change in sensation (i.e., burning, tenderness, more intense pricking). The borders were marked with a felt pen, and the rostral-caudal and lateral-medial distances were measured for later calculation of surface area using a rectangular model (length of longer side × length of shorter side).

### Statistical analysis

Data are presented as mean +/- SD (see figures). The primary outcome measure was the surface area of secondary hyperalgesia to both brush and von Frey hair simulation (measured at 40 minutes intervals for 6 hours after heat-capsaicin sensitization). The secondary measures included hot/cold pain thresholds and painfulness of the supra-threshold stimulation. The statistical comparison of the results (between placebo and valdecoxib pretreated) was performed using the two-tailed Wilcoxon signed ranks test. The calculation of sample size was based on a power calculation using our own preliminary data indicating that a minimum number of 20 volunteers is required to achieve 80% power (type II error at 0.2) to detect a clinically relevant change of 30% or more in the area of secondary hyperalgesia with alpha = 0.05 (paired, two-tailed test). A p-value equal to or less than 0.05 was considered statistically significant.

## Results

### Measurement of the success of blinding

At the end of each study session, 45% of the valdecoxib recipients and 20% of the placebo recipients correctly identified their group assignment (p = 0.092, Chi-square test).

### Pain threshold measurements

**Figure 1:**
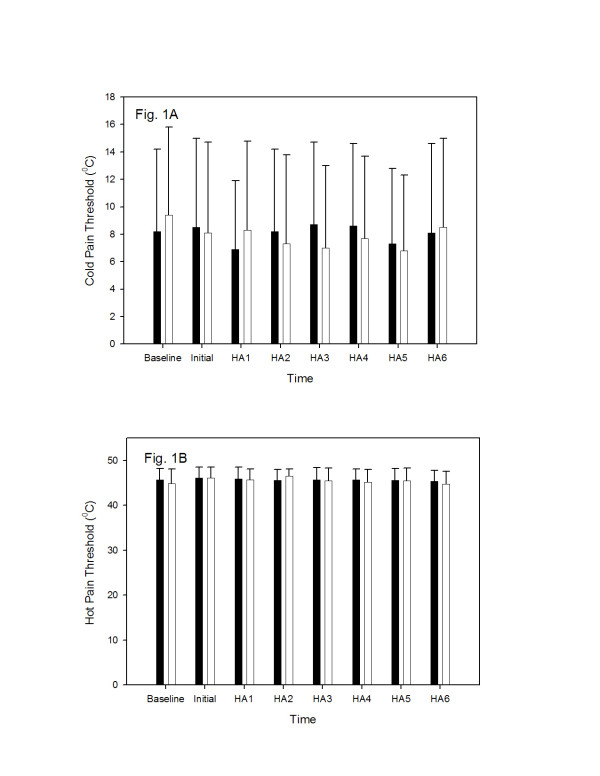
The effect of valdecoxib (40 mg, PO) and placebo on cold- (panel A) and hot (panel B) pain threshold. The pain threshold is expressed in °C (mean ± SD). Time scale indicates the time (in min) after study drug or placebo administration and when the pain measurements were performed. Black columns represent valdecoxib administration; white columns represent placebo administration. The results were compared using the Wilcoxon signed ranks test.

**Figure 2:**
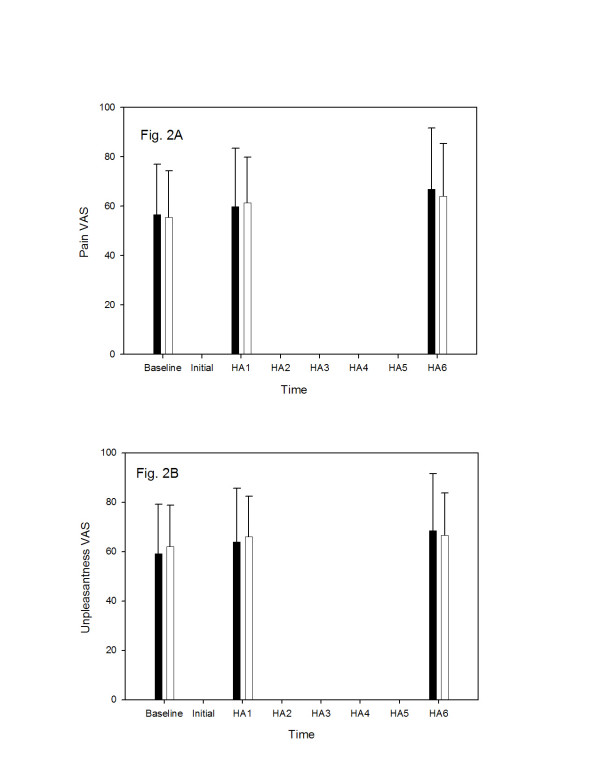
The effect of valdecoxib (40 mg, PO) and placebo on algosity (panel A) and unpleasantness (panel B) of the supra-maximal pain stimulus. The pain intensity is expressed in the visual analog scale of pain units (0 = no pain, 100 = maximum pain) as mean ± SD. Time scale indicates the time (in min) after study drug or placebo administration and when the pain measurements were performed. Black columns represent valdecoxib administration; white columns represent placebo administration. The results were compared using the Wilcoxon signed ranks test.

The results of the cold- and warm-threshold are presented in Figure 1A and 1B. No differences in the baseline cold-pain threshold were noted in the study volunteers. The mean cold-pain threshold did not significantly differ between the placebo- and valdecoxib-pretreated groups at any time (160–400 minutes) following administration of study drug (Figure 1A). Similarly, no differences in the baseline hot-pain threshold were observed either before administration of valdecoxib/placebo or at any time thereafter (Figure 2B).

### Painfulness of the supra-threshold stimulation

Baseline visual analog scores for pain algosity (Fig. 2A) and pain unpleasantness (Fig. 2B) were not statistically different at baseline or at either investigated time (1 and 6 hours) after valdecoxib or placebo administration.

### Heat-capsaicin sensitization method

**Figure 3:**
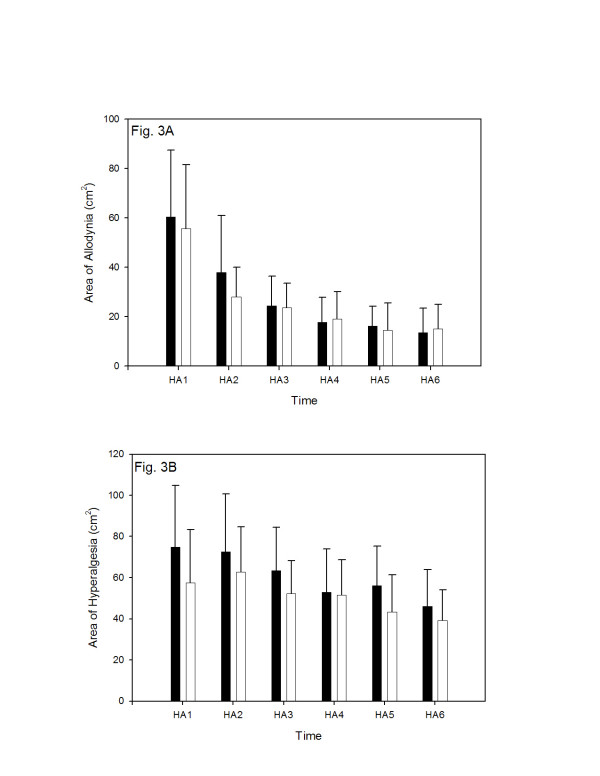
The effect of valdecoxib (40 mg, PO) and placebo on the skin area of allodynia (panel A) and mechanical hyperalgesia (panel B) after skin heat/capsaicin sensitization performed 120 min after study drug or placebo administration. The areas of allodynia (response to paint brush stimulation) and mechanical hyperalgesia (response to von Frey hair stimulation) are expressed as mean ± SD in cm^2^. Time scale indicates the time (in min) after study drug or placebo administration and when the pain measurements were performed. Black columns represent valdecoxib administration; white columns represent placebo administration. The results were compared using the Wilcoxon signed ranks test.

The heat-capsaicin sensitization method produced a large area of secondary hyperalgesia around the area of primary hyperalgesia (skin area directly stimulated by the thermode). The area of secondary hyperalgesia was characterized by allodynia (pain response to a previously non-noxious stimulus) and hyperalgesia (increased pain response to a noxious stimulus). Although rekindling maintained an area of secondary hyperalgesia, its size gradually diminished to approximately 50% of its initial size after five re-kindling sessions. This decline in size was more pronounced for brush hyperalgesia (allodynia) than for mechanical hyperalgesia using the von Frey hair. The area of allodynia (sensitivity to brush stimulation) was not statistically different between placebo and valdecoxib administration sessions (p > 0.05, Wilcoxon signed ranks test) at any time (160–400 minutes) (see Fig. 3A). Similarly, the area of hyperalgesia (sensitivity to von Frey hair stimulation) was not significantly different between placebo or valdecoxib administration sessions (p > 0.05, Wilcoxon signed ranks test) (Fig. 3B). The post hoc calculation of the power of the study, based on expected level of significance (P = 0.05), and the sensitivity threshold to detect a 30% reduction in the areas of mechanical hyperalgesia, revealed power values of 0.851, 0.929, 0.9756, 0.9665, 0.9564 and 0.9643 for all measurements taken between 160 and 400 min (i.e., at HA1 through HA6, respectively) after study drug or placebo administration. In contrast, when the post hoc power calculations were performed for the brush allodynia measurements, the sufficient power values (i.e., power value more than 0.8) were demonstrated only for the measurements taken at HA1 (160 min), HA2 (200 min) and HA3 (240 min) study drug or placebo administration (power values of 0.815, 0.805, 0.801, respectively).

### Adverse events

All 20 volunteers completed the study. The subjects tolerated the study medication and experimental procedures, and no adverse events were noted.

## Discussion

The main goal of the study was to answer the question whether an administration of the COX-2 inhibitor will reduce responses to heat/capsaicin induced hypersensitivity in humans. We showed that the administration of 40 mg of valdecoxib had no effect on skin sensitization in the heat-capsaicin model. Furthermore, valdecoxib had no analgesic effect following thermal/chemical sensitization (i.e., thermal pain threshold, intensity of the supra-threshold heat stimulation), when compared to baseline or placebo. The observed results were obtained during the period of time corresponding to the recently reported maximal plasma and cerebral spinal fluid, concentration after single 40 mg oral dose of valdecoxib [9]. It was also demonstrated that the single oral dose of 40 mg produced maximum plasma concentrations at 2 hrs after administration and lasting virtually undiminished (75% of maximum plasma concentration) until 6 hours post-administration [9]. In addition, the same authors demonstrated that valdecoxib produced maximum concentrations within the cerebrospinal fluid at 2 hours after administration, which lasted unchanged for another 6 hours. These results indicate that the pain measurements employed in our study were performed during the time of maximum concentrations of valdecoxib in both plasma and the central nervous system.

The current study is negative, which indicates either that the drug is ineffective or that the test is insensitive. The only way to demonstrate internal sensitivity of the test would have been to introduce a positive control. Not having the positive control included, we performed post-hoc calculations in order to demonstrate the power of the study regarding secondary hyperalgesia. The calculated power of the study exceeded 0.8 with data collected from 20 study volunteers (in paired design) for both measured components of the hyperalgesia response for at least initial half of all measurements (i.e., at HA1 through HA3), based on expected level of significance (P = 0.05), and the sensitivity threshold to detect a 30% reduction in the areas of hyperalgesia. In other words, the probability of type II error (i.e., not being able to demonstrate the presumable difference between placebo and valdecoxib) was less than 20%. It has been shown that an identical QST and heat-capsaicin model (including in many cases identical apparatus, thermode and the study protocol) had sufficient sensitivity in human volunteers to demonstrate the analgesic effects of intravenous infusion of low-doses of alfentanil [12], ramifentanil [14,16], lidocaine [17], intrathecally [12], but not systemically [18], administered adenosine, as well as orally administered gabapentin [11]. On the other hand, Mikkelsen et al. failed to demonstrate the analgesic efficacy of intravenous magnesium using a similar technique [13], and they reported that the effect of lidocaine was very modest [17]. Other investigations have demonstrated a correlation between the pre- and post-operative pain thresholds [18,19], using QST as well as for the prediction of the analgesic outcome after 3 steroid injections in sciatica [20]. Taken together, we can assume that the combined QST/heat-capsaicin method is sufficiently specific and sensitive for evaluation of analgesia in healthy volunteers. It is, however, possible that the level of sensitivity obtained by using this technique (i.e. 30% reduction in the surface area) is insufficient to detect the minimal analgesic effects of oral valdecoxib on secondary hyperalgesia.

Previous animal studies suggest that COX2 inhibitors may have different effect on the development of the secondary hypersensitivity, depending on the drug [21,22] and experimental model [23]. There is evidence that topical COX2 inhibition is effective in capsaicin pain model in humans [24]. The results of our study seem to partially contradict the above-presented data. On the other several animal studies demonstrated lack of efficacy of COX inhibitors against acute noxious heat stimuli in the normal condition (i.e., without previous sensitization [25-27]. These studies would lead one to predict that this COX-2 inhibitor would have no effect on acute thermal heat responses, either threshold or supra-threshold (as was observed), but that it might have efficacy against hypersensitivity. The negative results in this study should be therefore attributed rather to experimental design, including experimental model, but also the use of pretreatment, which does not allow observing changes from the untreated baseline during placebo session. On the other hand it is important to note that a similar lack of effect of valdecoxib to prevent visceral pain hypersensitivity in healthy volunteers was demonstrated recently in a validated human esophageal pain hypersensitivity model [28]. Finally, it was demonstrated previously, that the main cause of the variability in pain trials might be random chance associated with the relatively small size of the trial group [29]. It is therefore possible, that the results of this study, which contradicts clinical experience and trial results in clinical pain states, might be due to the random play of chance, and us such need to be confirmed in larger studies or from pooling multiple trials of conventional (small) size).

## Conclusion

We demonstrated in a double-blind, cross-over, prospective study that a single, orally administered dose of valdecoxib does not attenuate secondary hyperalgesia/allodynia induced by the skin heat/capsaicin sensitization model, when compared to placebo.

## Competing interests

The study was supported by the Independent Investigator Grant from the manufacturer of valdecoxib – Pfizer, Inc.

## Authors' contributions

DB participated in study design, and collected and analyzed the clinical data.

LH collected the clinical data, participated in analysis and interpretation of data

MH collected the clinical data, participated in analysis and interpretation of data

TMJ participated in the procedure, involved in drafting the manuscript

GS participated in the procedure, involved in drafting and revising the manuscript

PKJ conceived and designed the study, processed the data and wrote the manuscript.

All authors read and approved the final manuscript.

## Pre-publication history

The pre-publication history for this paper can be accessed here:


